# In and out among the Hospitals

**Published:** 1888-07-14

**Authors:** 


### In and Out Among the Hospitals.

By a Secretary of Ten Years' Standing.

Copies of Annual Reports and Rules, Accounts of Meetings, Changes in the Staff, &c., should be addressed The Editor, The Lodge, Porchester Square, W.

### ST. GEORGE'S HOSPITAL.

A great improvement has been effected at St. George's, the whole of the flooring of the hospital having been removed. The work has been done during the summer months of the last three years, and is now complete. The new floors, which are of teak, are a great improvement on the old deal boards; considerable alterations have also been made in the out-patients' department. These works have, of course, greatly reduced the number of patients treated: the daily average last year was only 277 in-patients, and the number of out-patients 22,410. The receipts were £24,952, and the expenditure £27,508, this latter including £4,577 for extraordinary expenses. Deducting this and 1*s.* for each out-patient, the cost per bed occupied was £78 12*s.* 5*d.* As soon as patients can be moved, they are drafted off to the Atkinson Morley Convalescent Home at Wimbledon, and this therefore reduces the stay of the patients in hospital: over 30 per cent. of the patients treated in the wards were sent to Wimbledon last year. Dr. Wadham and Mr. Timothy Holmes have both completed their period of office of twenty years as physician and surgeon, and have been respectively appointed consulting physician and surgeon. The vacancies that have thus been caused on the staff have been filled up by the appointment of Dr. Gamgee, F.R.S., and Mr. Turner. Nearly all the trust funds of the hospital have been put in the hands of the official trustees of charitable funds. This system of placing trust money with the Charity Commissioners is much to be commended. All difficulty as to change of trustees is thus avoided, and there is no danger of bequests left for a definite purpose being absorbed in the general funds of the hospital.

### THE SUFFOLK GENERAL HOSPITAL.

At this county hospital the number of patients treated in the wards last year was 391, and there were besides 1,092 out-patients. The number of in-patients is below the average, owing to two of the wards having been closed for repairs. The daily average number of beds occupied was only 47. The total receipts for the year amounted to £2,780 12*s.*, an increase of £54 on the preceding twelve months, although there was a falling off in subscriptions and church collections. There was a total expenditure of £2,768 17*s.* 2*d.*, of which a large sum was expended in repairs; but it is curious to note that there was an increase of £14 15*s.* 2*d.* in the household expenses, notwithstanding that the number of patients under treatment was considerably less. After deducting 1*s.* for each out-patient, the cost per bed occupied is £57 15*s.* To commemorate the Queen's Jubilee the committee appealed for funds "for facing the walls of the two lower wards with tiles, and to form a retiring fund for nurses." The appeal produced the sum of £487 2*s.* 6*d.*, out of which £482 0*s.* 5*d.* was expended on the tiles and the balance £5 2*s.* 1*d.*, was credited to the nurses' proposed Pension Fund. The last-mentioned sum will not go far towards providing pensions, &c., and the division of the collection seems a little extraordinary. It may have been considered now that the nurses can join the National Pension Fund for Nurses, the very smallest portion possible was enough to satisfy those who are in favour of establishing a special pension fund in the Suffolk Hospital. At the last general meeting three names were added to the list of vice-presidents by the election of R. Macnab, Esq., M.D., W. E. Image, Esq., and J. G. Barclay, Esq. The late matron (Mrs. Robinson), who was elected in 1884, resigned on May 17th last. Under her management many changes and improvements were introduced tending to increase the comfort of the patients and nurses. Miss Shaw was elected to fill the vacancy thus created.

### QUEEN'S HOSPITAL, BIRMINGHAM.

This, one of the best-managed hospitals in the provinces, has again to record a prosperous year's work. The report before us is concise and excellently compiled; the accounts are clear, and are set forth in a manner that can be understood by the most inexpert, yet at the same time are prepared accurately and in a way that would be passed by the most critical accountant. The greater part of the difficulty felt by governors and the uninitiated in understanding hospital finance would vanish if committees would publish their reports on the lines of this of the Queen's Hospital. The excess of income over expenditure was £63, which sum has gone to reduce an old debt of £977. The income for the year was £7,690, the daily average number of beds occupied, 113. Taking 27,230 out-patients at 1*s.* each, and deducting this sum from the expenditure, the cost per bed occupied was £55 9*s.* This, in a large manufacturing town, where most necessaries are as dear as in London, is a lesson to many metropolitan hospitals. The workmen of Birmingham have subscribed the handsome sum of £1,115. This spring extensive alterations were begun, the cost of which will be defrayed by a special fund. The estimated amount required is £10,000, the greater portion of which has been already subscribed. The improvements include the placing of the lavatories, &c., in towers outside the building, and the removal of the chapel, which will give additional space for beds; besides this, new floors will be laid and additional accommodation supplied for the nurses. The following changes have occurred on the staff: Mr. F. Marsh, F.R.C.S., was elected surgeon, vice Mr. F. Jordan, resigned; and Mr. J. T. J. Morrison, F.R.C.S., appointed casualty surgeon, in place of Mr. J. Marsh. It seems strange that the same officer should occupy the post of obstetric and ophthalmic house surgeon.

### WESTMINSTER HOSPITAL.

During last year the Westminster Hospital benefited perhaps more than any other charity, by the Queen's Jubilee. Its proximity to the Abbey and St. Margaret's naturally causes a great interest in its work to be taken by the Dean of of Westminster and the other clergy, and this interest assumed such substantial form, that altogether over a thousand pounds was handed to the funds, besides £2,712, which was realised by letting seats at the hospital to view the Jubilee procession. The total receipts were £17,528, and the expenditure £16,718. Deducting from this latter sum the cost of out-patients at 1*s.* per head and expenses on new buildings, the cost per bed occupied is £65 11*s.* 5*d.* The daily average number of patients was 172. An accurate account has been kept by the committee of the expenditure on out-patients, and in a carefully compiled table is shown the cost for the last three years. Taking the average of this period and the average of out-patients, it is found that each out-patient cost 1*s.* 2½*d.* (Out-patients are estimated at 1*s.* each in writing these papers for the sake of comparing one hospital with another.) The committee take a considerable interest in the medical school, and lately encouraged it by granting an entrance scholarship of £20 per annum from the funds of the hospital. The medical school report announces that there was a considerable increase in the number of students. There appears to have been few changes on the medical staff. Mr. A. M. Sheild resigned the office of assistant surgeon, and Mr. Charles Stoneham was elected to the vacant office. It has also been decided to increase the surgical staff by the appointment of a fourth assistant surgeon, and Mr. Walter G. Spencer has been elected to this office. The nursing staff continues to give entire satisfaction. It was resolved that a few nurses should attend daily in the out-patient department, an arrangement which has worked very well in practice, and is a great boon to the patients, especially to women and children. The committee have adopted the following table for the endowment of beds and cots: Perpetual endowment, bed £1,000, cot £600; endowment during life of donor, £400, cot £250; supported by annual donation, bed £50, cot £30. So far the hospital has been successful in getting ten beds and five cots endowed.

